# Urinary Angiopoietin-2 Is Associated with Albuminuria in Patients with Type 2 Diabetes Mellitus

**DOI:** 10.1155/2015/163120

**Published:** 2015-03-19

**Authors:** Shan Chen, Huiqing Li, Chun Zhang, Zhenqiong Li, Qiuyuan Wang, Jinting Guo, Changqing Luo, Yumei Wang

**Affiliations:** ^1^Department of Nephrology, Union Hospital, Tongji Medical College, Huazhong University of Science and Technology, No. 1277 Jiefang Avenue, Wuhan, Hubei 430022, China; ^2^Department of Endocrinology, Union Hospital, Tongji Medical College, Huazhong University of Science and Technology, Wuhan, Hubei 430022, China

## Abstract

*Aims*. To evaluate the levels of angiopoietin-1 (Ang-1), Ang-2, and vascular endothelial growth factor (VEGF) in serum and urine, and their association with albuminuria in patients with type 2 diabetes mellitus. *Methods*. In 113 type 2 diabetic patients with normoalbuminuria, microalbuminuria, and macroalbuminuria and 30 healthy controls, the levels of Ang-1, Ang-2, and VEGF in serum and urine were measured by enzyme-linked immunosorbent assay (ELISA). *Results*. Urinary and serum levels of Ang-2 were significantly higher in diabetic patients with normoalbuminuria than in healthy controls. Increased urinary Ang-2 level was positively associated with the degree of albuminuria. Urinary Ang-1 levels were significantly higher in normoalbuminuria patients and lower in macroalbuminuria patients than in controls. The levels of urinary VEGF increased in the albuminuria subgroup, though serum levels of Ang-1 and VEGF did not change. Urinary Ang-2 levels were correlated positively with albuminuria and negatively with glomerular filtration rate (GFR). Stepwise multiple regression analysis identified albuminuria (*P* < 0.001) and GFR (*P* = 0.001) as significant predictors of urinary Ang-2. *Conclusions*. Our data suggest that urinary Ang-2 is stepwise increased with renal damage in patients with type 2 diabetes mellitus and is associated with albuminuria.

## 1. Introduction

Diabetic nephropathy (DN) is a common complication of type 2 diabetes mellitus (DM) and is the leading cause of end-stage renal disease (ESRD). Microalbuminuria is an early sign of DN, which correlates with and can predict the progression of renal damage and cardiovascular morbidity [1–5]. The discovery of biomarkers for the earlier stages of DN would enable early intervention to reduce the impact of this chronic vascular complication.

Diabetic nephropathy is associated with altered vascular structure, endothelial dysfunction, and disrupted homeostasis and angiogenesis [4, 6, 7]. Abnormal glomerular angiogenesis in patients with DN is associated with glomerular hypertrophy and results in glomerular capillary injury and urinary albumin excretion [8, 9]. Thus, the mechanisms for the development of abnormal angiogenesis in DN involve a complicated interplay between pro- and antiangiogenic factors. Two families of growth factors, angiopoietin/Tie-2 and vascular endothelial growth factor (VEGF)/VEGF receptor (VEGFR), are thought to be associated with the development of DN. VEGF increases vascular permeability and is mitogenic for endothelial cells, acting early and at most points of the angiogenic cascade [10]. Within the angiopoietin family, angiopoietin-1 (Ang-1) and angiopoietin-2 (Ang-2) are the best-studied ligands for Tie-2 receptors. Ang-1 signaling via Tie-2 is involved in capillary sprouting, endothelial cell survival, and vascular remodeling [11]. Ang-2 is a natural antagonist of Ang-1 [12, 13]. Several studies suggest that Ang-2 signaling, in combination with VEGF, leads to sprouting angiogenesis, while Ang-2 signaling in the absence of VEGF causes vessels to regress [11]. Hence, selective upregulation of VEGF and Ang-2 may lead to aberrant proliferation of leaky, friable vessels [10]. Emerging evidence suggests that VEGF and angiopoietin are critical in glomerular physiology and in the pathogenesis of glomerular disease in DM [14–17].

Previous studies have reported upregulated plasma levels of VEGF and Ang-2 in human and animal DN [10, 16, 18–20]. Notably, circulating Ang-2 levels associated with albuminuria have been reported in chronic kidney disease (CKD) [5] and systemic lupus erythematosus (SLE) [21]. Transgenic mice with inducible overexpression of Ang-2 in podocytes have been shown to develop significant increases in albuminuria [22], which, in turn, correlates with and predicts the progression of renal damage in DM [23]. Increased Ang-2 levels in patients with DM are associated with indexes of endothelial damage and dysfunction [16]. However, little is known about the urinary levels of these angiogenic factors in different stages of DN, and potential correlations between them and albuminuria have not yet been studied. In the present study, we measured urinary Ang-2, Ang-1, and VEGF levels to elucidate the possible correlation between urinary angiogenic factors and renal damage in patients with various phases of type 2 DM.

## 2. Materials and Methods

### 2.1. Subjects

The retrospective study included 113 insulin-dependent patients with type 2 DM recruited from the Department of Endocrinology and Nephrology at Union Hospital (Wuhan, China) between December 2012 and March 2014. Type 2 DM was defined according to established WHO criteria [24] (i.e., fasting blood glucose (FBG) ≥ 7.0 mmol/L, postprandial blood glucose ≥ 11.1 mmol/L, or symptoms of DM with random blood glucose ≥ 11.1 mmol/L). The mean duration of DM was 10.33 years (1 month–30 years). All patients maintained a stable body weight for at least 3 months before beginning the study. None of the patients had evidence of acute diabetic complications. Patients with acute vascular events or hospitalization (defined as stroke, myocardial infarction, unstable angina, or coronary or peripheral revascularization within the last 3 months) [16]; high-range hypertension (≥160/100 mmHg); current infection; or evidence of neoplastic, hepatic, or significant renal disease (requiring dialysis) within 3 months prior to enrollment were excluded from the study [16]. Patients requiring treatment with glucocorticoids or other drugs affecting glucose metabolism were also excluded. Diagnosis of DN was made according to the criteria of Kidney Disease Outcomes Quality Initiative (KDOQI) [25]. Based on the urinary albumin excretion rate (UAER) at baseline, patients were classified as having normoalbuminuria (DN1 group: UAER < 20 *μ*g/min), microalbuminuria (DN2 group: UAER 20–200 *μ*g/min), or macroalbuminuria (DN3 group: UAER > 200 *μ*g/min). Thirty subjects undergoing routine health checks were recruited into the control group (NC). The study protocol was approved by the Medical Ethics Committee of Union Hospital, Tongji Medical College, Huazhong University of Science and Technology, and was conducted according to the principles of the Declaration of Helsinki. Patients and control subjects provided written informed consent to participate in the study.

### 2.2. Laboratory Measurements

Morning preprandial levels of fasting blood sugar (FBG), serum creatinine, triglyceride (TG), high-density lipoprotein cholesterol (HDL-ch), low-density lipoprotein cholesterol (LDL-ch), and total cholesterol were measured with a full automatic biochemical analyzer (Hitachi 7150, Tokyo, Japan). Glycosylated hemoglobin (HbA1c) was measured with a D10 hemoglobin testing system (Bio-Rad Laboratories, Hercules, CA, USA), using a cation exchange HPLC and an immunoturbidimetric assay method (Roche/Hitachi 902 Cobas System). Twenty-four h urine samples were collected for the determination of Ang-1, Ang-2, and VEGF levels and the estimation of UAER. Urinary protein quantitative measurements (24 h UPQM) were obtained for all patients. Renal function was assessed by glomerular filtration rate (GFR) detection with single photon emission computed tomography (SPECT) (GE Millennium MG Dualhead, GE Healthcare, Milwaukee, WI, USA, USA). Vital signs and body mass index (BMI) were recorded.

### 2.3. Quantification of Ang-1, Ang-2, and VEGF

Levels of angiogenic factors (Ang-1, Ang-2, and VEGF) in serum and urine were measured by enzyme-linked immunosorbant assay (ELISA) in frozen serum samples and in urine samples. In brief, monoclonal antibodies specific for Ang-1 (Raybiotech, Norcross, GA, USA), Ang-2 (Raybiotech), and VEGF (NeoBioscience Technology Co., Shenzhen, China) were precoated onto microplates. Standards and samples were pipetted into the wells. Ang-1, Ang-2, or VEGF present in a sample was bound to the wells by the immobilized antibody. The wells were washed, and biotinylated anti-human antibody specific for Ang-1, Ang-2, or VEGF was added. After the removal of unbound antibodies, HRP-conjugated streptavidin was pipetted into the wells. The wells were washed, and a 3,3′,5,5′-tetramethylbenzidine (TMB) substrate solution was added. The TMB solution changed in color from blue to yellow, in proportion to the bound concentration of Ang-1, Ang-2, or VEGF. The absorbance of the solution in each well was measured by a microplate reader (Bio-Tek ELx800; VT, USA) at a wavelength of 450 nm. All samples were examined in duplicate, and mean values were used for statistical analysis.

### 2.4. Statistical Analysis

Statistical analysis was performed using a commercially available statistical software package (SPSS for Windows, version 18.0; SPSS, Chicago, IL, USA). Data were presented as the mean ± SEM. One-way analysis of variance (ANOVA) with Tukey's post hoc test was used to compare groups of normally distributed data. Nonnormally distributed data were analyzed using the Kruskal-Wallis test. Categorical data were analyzed using the Chi-square test, and Pearson's or Spearman's correlation coefficients were used to test associations between variables. A stepwise multiple regression analysis was performed to identify the predictors of Ang-2 (dependent variable). All tests were two tailed, and values of *P* < 0.05 were considered statistically significant.

## 3. Results

### 3.1. Patient Characteristics

The clinical and biochemical characteristics of the study subjects are shown in Table 1. No significant differences were found in age, gender, BMI, diastolic blood pressure (DBP), total cholesterol, LDL-ch, HDL-ch, or TG among the three diabetic groups and the control group. However, systolic blood pressure (SBP), HbA1c, and FBS were higher in the diabetic patients than in control subjects. HbA1c appeared to be lower in the DN3 group than in the DN1 and DN2 groups after long-term treatment with hypoglycemic drugs; however, no significant differences in HbA1c and FBS were found among the three diabetic groups. Serum creatinine was significantly higher, and GFR was significantly lower, in the DN3 group than in the DN1 and DN2 groups (*P* < 0.001). Both UAER and 24 h UPQM increased progressively from the DN1 to the DN3 groups (*P* < 0.001).

### 3.2. Angiogenic Growth Factors in Serum and Urine

**Figure 1:**
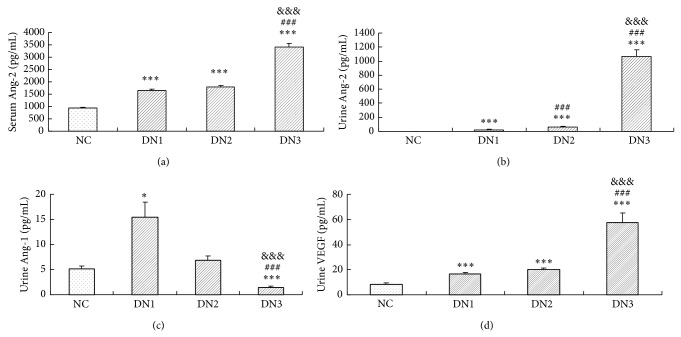
Serum and urinary angiogenic growth factor levels in diabetic patients and controls. (a) Statistical analysis showed increased serum concentrations of Ang-2 in diabetic patients compared with controls. (b) The level of urinary Ang-2 showed a stepwise increase in diabetic patients compared to controls according to the degree of albuminuria. (c) Urinary Ang-1 level was significantly higher in the DN1 group and lower in the DN3 group when compared with control subjects. Patients in the DN1 and DN2 groups exhibited significantly higher urinary Ang-1 levels than those in the DN3 group. (d) Subjects with diabetes mellitus showed significantly higher urinary VEGF levels than control subjects. Patients were divided into DN1 (normal-albuminuria), DN2 (microalbuminuria), and DN3 (macroalbuminuria) groups. ^*^ *P* < 0.05* versus* NC; ^***^ *P* < 0.001* versus* NC; ^###^ *P* < 0.001* versus* DN1; ^&&&^ *P* < 0.001* versus* DN2.

Serum levels of Ang-2 were markedly increased in diabetic patients compared with values in the control group (*P* < 0.001; Figure 1(a)). Moreover, serum Ang-2 was significantly higher in patients with macroalbuminuria (DN3) than those in the DN1 and DN2 groups (*P* < 0.001; Figure 1(a)). Diabetic patients exhibited higher levels of urinary Ang-2 than controls (*P* < 0.001; Figure 1(b)), and urinary Ang-2 increased in a stepwise manner with increasing degrees of albuminuria in the three diabetic groups (*P* < 0.001; Figure 1(b)).

No significant difference was found in serum Ang-1 levels between any of the four groups (data not shown). However, urinary Ang-1 levels were significantly higher in the DN1 group than in the control group (*P* < 0.05; Figure 1(c)) and lower in the DN3 group than in the control group (*P* < 0.001). In addition, patients in the DN1 and DN2 groups had significantly higher urinary Ang-1 levels than those in the DN3 group (*P* < 0.001; Figure 1(c)).

No significant difference was found in serum VEGF among the groups (data not shown); however, subjects with DM exhibited significantly higher urinary VEGF levels than the control subjects (*P* < 0.001; Figure 1(d)). Moreover, urinary VEGF was significantly higher in patients with macroalbuminuria (DN3) than in the DN1 and DN2 groups (*P* < 0.001; Figure 1(d)). No difference was observed in urinary VEGF in diabetic patients with or without microalbuminuria.

### 3.3. Correlation and Multivariate Analysis

**Figure 2:**
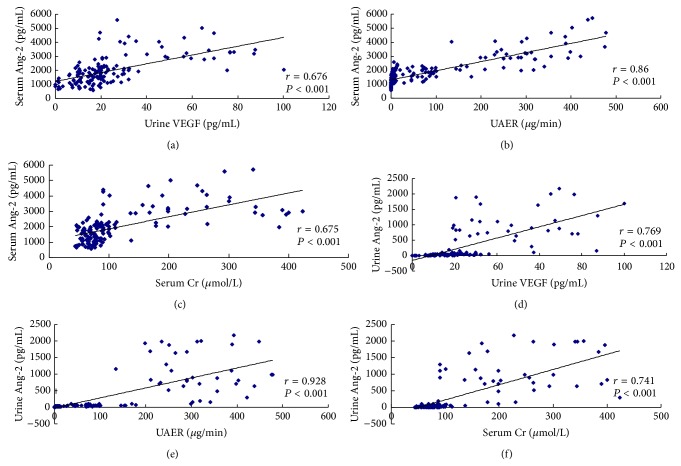
Correlation analysis for serum Ang-2 and urinary Ang-2 with urinary VEGF, UAER, and serum creatinine. Serum Ang-2 level correlated positively with urinary VEGF (a), UAER (b), and serum creatinine (c). Similarly, Urinary Ang-2 correlated positively with urinary VEGF (d), UAER (e), and serum creatinine (f). Ang-2: angiopoietin 2; VEGF: vascular endothelial growth factor; UAER: urinary albumin excretion rate; Cr: creatinine.

Table 2 and Figure 2 summarize the results of the analyses undertaken in patients with DN. Serum levels of Ang-2 were significantly positively correlated with urinary Ang-2 and VEGF levels (all *P* < 0.001), as well as with UAER, 24 h UPQM (both *P* < 0.001), and serum creatinine (*P* < 0.001). In addition, serum Ang-2 was negatively correlated with GFR (*P* < 0.001).

Similarly, urinary Ang-2 was strongly correlated with urinary VEGF, UAER, 24 h UPQM, and serum creatinine (all *P* < 0.001). A negative correlation was found between GFR and urinary Ang-2 (*P* < 0.001). No significant correlations were found between serum or urinary Ang-2 and age, BMI, DBP, SBP, HbA1c, FBS, HDL-ch, LDL-ch, total cholesterol, or triglyceride (data not shown). In these subjects, stepwise multiple regression analyses including UAER, GFR, age, BMI, DBP, SBP, FBS, HbA1c, and serum Ang-2 identified that UAER (*P* < 0.001) and GFR (*P* = 0.001) are significant predictors of urinary Ang-2.

## 4. Discussion

This study is the first to investigate urinary levels of Ang-1, Ang-2, and VEGF in human type 2 DM with varying UAERs. We found the following. (1) Urinary Ang-2 increased in a stepwise manner in type 2 DM patients with various degrees of kidney damage (normoalbuminuria, microalbuminuria, and macroalbuminuria). This alteration was accompanied by increased urinary VEGF, as well as early increased and later decreased urinary Ang-1. (2) Urinary Ang-2 levels in normoalbuminuria patients increased prior to changes in albumin levels. (3) Among the angiogenic growth factors, urinary Ang-2 was strongly associated with degree of albuminuria and GFR in type 2 DM patients.

Alterations in the VEGF and Ang-1/Ang-2 system have been reported to play an important role in the pathobiology of glomerular disease in DM [2, 14–17, 20, 26] and chronic kidney disease (CKD) [5, 27–29]. Most of those studies focus on circulating levels of angiogenic growth factors. In this study, we evaluated simultaneously the levels of Ang-1, Ang-2, and VEGF in both serum and urine. We found that urinary Ang-2 increased stepwise with albuminuria levels than Ang-1 and VEGF at a greater rate. In addition, serum and urinary Ang-2 are increased in normoalbuminuric patients. This observation is likely due to the tubular pathophysiological changes, which occur before the glomerular stage of disease. This suggests that the serum and urinary Ang-2 are related to subclinical tubular impairment and may be an earlier measurable marker of renal involvement before the onset of albuminuria.

Increasing evidence suggests that upregulation of Ang-2 is pathologically harmful to the kidney [2]. Ang-2 has been linked to increased microvascular permeability [30], and podocyte overexpression of Ang-2 was shown to produce albuminuria in transgenic mice [22]. Circulating Ang-2 levels correlating positively with proteinuria have been reported in human SLE [21] and CKD [5]. In our type 2 DM patients, we found that albuminuria is a significant predictor for urinary Ang-2 levels after adjustments were made for those possible confounders. The association between urinary Ang-2 and albuminuria suggests that upregulation of Ang-2 may destabilize glomerular endothelial cells and directly or indirectly affect podocytes, leading to the deterioration of glomerular filtration barrier function [5, 31]. Abundant Ang-2 protein was detected by immunohistochemical staining in glomeruli—in endothelial cells alongside capillary loops—in renal biopsies from patients with DM (data not shown). Taken together, these findings suggest that high glucose-induced glomerular endothelial damage may lead to secretion of Ang-2, and elevated expression of Ang-2 in the glomerular endothelium may further increase albuminuria through the damaged glomerular filtration barrier [17]. This possibility requires further investigation.

The positive correlation with serum creatinine and the negative correlation with GFR suggest that increased Ang-2 may be associated with the development of renal impairment. Blood Ang-2 levels rise in line with the decline in renal function in type 2 DM [32] and CKD [29], and this inverse correlation may predict long-term mortality in patients with CKD [27, 33]. In our study, we found that urinary Ang-2 level is inversely related to GFR. These observations suggest that urinary Ang-2 levels increase in parallel to the deterioration of renal function [29]. In our study, elevated urinary Ang-2 was correlated with serum Ang-2, suggesting that urinary levels of Ang-2 may be representative of local production and release of Ang-2 into the circulation in patients with DN. Alternatively, as DN progresses, decreased GFR leads to higher serum Ang-2, which allows greater penetration of the glomerular barrier and leads to proteinuria. However, the multivariate analysis showed that serum Ang-2 is not a predictor for urinary Ang-2. The correlation between serum and urinary Ang-2 requires further study.

Based on the correlation between serum Ang-2 and HbA1c, accumulation of advanced glycation end product (AGE) in endothelial cells subjected to hyperglycemia may upregulate serum levels of Ang-2 and VEGF [34, 35]. However, no correlation was found between urinary and/or serum Ang-2 and HbA1c in our study. The differences between studies may reflect variations in study design. However, the possibility can be not excluded that increased urinary Ang-2 levels are a consequence of mechanisms that are unassociated with the accumulation of AGEs in the glomerular endothelium.

We unexpectedly found that urinary Ang-1 was significantly higher in DM patients with normoalbuminuria and lower in those with macroalbuminuria than in control subjects. This observation, also reported by Rizkalla, showed upregulation of Ang-1 in the early phase of the disease and progressive downregulation of renal Ang-1 expression in experimental DM [17, 26]. Ang-1 is produced by glomerular podocytes [36, 37] and plays an important role in maintaining the structure and integrity of the glomerular filtration barrier [38]. Increased urinary Ang-1 in DN1 patients compared to control is a novel finding. Further studies will seek to determine whether this increase is a short-term response of podocytes to hyperglycemia or will reduce the vascular permeability through endothelial cell glycocalyx layer [39] modifications or other mechanisms. The decrease in Ang-1 in subjects with macroalbuminuria suggests that Ang-1 production is attenuated at the later stage of DN, which may be associated with a decreased number or function of podocytes, or both. Of course, this possibility requires further investigation. In our study, we found no differences in serum Ang-1 levels in groups with varying degrees of albuminuria. This observation is consistent with previously reported findings [16]. However, Dessapt-Baradez reported decreased Ang-1 levels in mice with streptozotocin-induced type 1 DM, which was accompanied by marked albuminuria, nephromegaly, hyperfiltration, glomerular ultrastructural alterations, and aberrant angiogenesis [38]. The differential expression pattern of Ang-1 in serum may be due to the different subjects and varying stage of DN.

Urinary VEGF was increased in our study in all diabetic groups—even at the normoalbuminuric stage—though serum VEGF was unaltered. These findings are in agreement with previously published findings [10, 14, 18, 40]. However, some centers have reported an increase in plasma VEGF in type 2 DM [19, 41, 42]. These differences likely reflect the different populations being studied, as well as variations in study design.

## 5. Conclusion

The results of this study show that urinary Ang-2 increases in a stepwise manner in type 2 diabetic patients with varying degrees of kidney damage. Urinary Ang-2 level is associated with albuminuria in type 2 diabetic patients. Ang-2 measurement in urine is a useful, noninvasive tool for the evaluation of renal involvement in the course of DM, especially in normoalbuminuric patients. Further investigations with a larger sample size and a prospective design are required to confirm the potential application of Ang-2 as a useful biomarker for the early detection of diabetic nephropathy.

## Figures and Tables

**Table 1:** Clinical and laboratory characteristics.

Group	NC	DN1	DN2	DN3	*P*
Number	30	38	37	38	—
Age (year)	50.9 ± 2.05	48.21 ± 1.73	51.38 ± 1.73	55.16 ± 1.84	>0.05
Male/female	17/13	23/15	20/17	21/17	>0.05
BMI (kg/m^2^)	22.99 ± 0.24	23.13 ± 0.32	23.89 ± 0.33	23.32 ± 0.33	>0.05
SBP (mmHg)	123.2 ± 1.76	119.95 ± 1.14	143.78 ± 1.56	150.11 ± 2.02	<0.001^*^
DBP (mmHg)	77.47 ± 0.85	80.08 ± 0.77	78.51 ± 0.91	79.92 ± 0.99	>0.05
Total cholesterol (mmol/L)	4.08 ± 0.1	4.47 ± 0.14	4.24 ± 0.15	4.57 ± 0.15	>0.05
LDL-ch (mmol/L)	2.72 ± 0.09	2.7 ± 0.09	2.65 ± 0.09	3.01 ± 0.16	>0.05
TG (mmol/L)	1.71 ± 0.11	1.92 ± 0.08	2.09 ± 0.13	1.8 ± 0.12	>0.05
HDL-ch (mmol/L)	1.26 ± 0.05	1.25 ± 0.04	1.24 ± 0.04	1.42 ± 0.07	>0.05
UAER (*μ*g/min)	0	6.98 ± 0.66	81.31 ± 6.22	338.895 ± 21.92	<0.001^*^
24 h UPQM (g/24 h)	0	0.099 ± 0.016	0.45 ± 0.03	4.17 ± 0.36	<0.001^*^
Serum Cr (*μ*mol/L)	67.56 ± 2.43	66.05 ± 1.9	89.18 ± 2.5	236.02 ± 15.89	<0.001^*^
GFR (mL/min)	87.89 ± 1.31	118.12 ± 2.66	104.07 ± 1.77	49.69 ± 2.84	<0.001^*^
HbA1c (%)	6.08 ± 0.09	9.02 ± 0.24	9.05 ± 0.27	7.49 ± 0.26	<0.001^*^
FBS (mmol/L)	5.17 ± 0.05	7.70 ± 0.19	7.5 ± 0.19	6.74 ± 0.22	<0.001^*^

Data are expressed as mean ± standard error of the mean (SEM). *P* values were estimated using analysis of variance (ANOVA) or the Kruskal-Wallis test.

NC: normal control; DN1: normal-albuminuria group; DN2: microalbuminuria group; DN3: macroalbuminuria; BMI: body mass index; Cr: creatinine; DBP: diastolic blood pressure; GFR: glomerular filtration rate; FBS: fasting blood sugar; HDL-ch: high-density lipoprotein cholesterol; LDL-ch: low-density lipoprotein cholesterol; SBP: systolic blood pressure; TG: triglyceride; UAER: urinary albumin excretion rate; 24 h UPQM: 24 h urinary protein quantitative measurements; HbA1c: glycosylated hemoglobin; ^*^significant difference between diabetic patients and controls.

**Table 2:** Correlations between potential markers of DN.

	Urine Ang-2	Serum Ang-1	Urine Ang-1	Serum VEGF	24 h UPQM	GFR	HbA1c
Serum Ang-2	*r* = 0.799	*r* = −0.022	*r* = −0.446	*r* = 0.999	*r* = 0.826	*r* = −0.326	*r* = 0.126
	(*P* < 0.001)	(*P* > 0.05)	(*P* < 0.001)	(*P* > 0.05)	(*P* < 0.001)	(*P* < 0.001)	(*P* > 0.05)

Urine Ang-2	—	*r* = 0.036	*r* = −0.406	*r* = 0.112	*r* = 0.936	*r* = −0.389	*r* = 0.17
		(*P* > 0.05)	(*P* < 0.001)	(*P* > 0.05)	(*P* < 0.001)	(*P* < 0.001)	(*P* > 0.05)

Data are presented as Pearson's or Spearman's correlation coefficients (*r*) and *P* values.

Ang-1: angiopoietin-1; Ang-2: angiopoietin-2; VEGF: vascular endothelial growth factor. For other abbreviations see Table 1.
